# Does Subjective Well-Being Promote Pro-Environmental Behaviors? Evidence from Rural Residents in China

**DOI:** 10.3390/ijerph19105992

**Published:** 2022-05-14

**Authors:** Xi Ouyang, Wen’e Qi, Donghui Song, Jianjun Zhou

**Affiliations:** 1 National School of Agricultural Institution and Development, South China Agricultural University, Guangzhou 510642, China; ouyangxi@stu.scau.edu.cn (X.O.); dhsong@stu.scau.edu.cn (D.S.); 2 College of Economics and Management, South China Agricultural University, Guangzhou 510642, China; 3 School of Economics, Central University of Finance and Economics, Beijing 100081, China; 2018110098@email.cufe.edu.cn

**Keywords:** subjective well-being, pro-environmental behavior, reciprocal, altruism, rural residents

## Abstract

The spontaneous pro-environmental behavior (PEB) of rural residents is essential for rural environmental governance. Existing studies have primarily focused on the impact of objective factors on individual PEB, while less attention has been paid to the role of subjective factors, such as rural residents’ subjective well-being, in shaping such behaviors. Based on the Chinese General Social Survey (CGSS) data, this study evaluates the impact of subjective well-being on the PEB of rural residents. The results show that subjective well-being significantly promoted the PEB in both the private sphere with reciprocity and the public sphere with altruistic attributes. Subjective well-being affected PEB mainly by enhancing rural residents’ social interaction and reciprocity with others and raising their fraternity and altruism. Moreover, the positive effect was mainly driven by women and individuals with more environmental knowledge. Therefore, enhancing rural residents’ subjective well-being is not only an important development goal, but also the starting point and foothold of solving the contradiction between economic development and environmental protection and promoting social harmony.

## 1. Introduction

Soil, water and air pollution, ecosystem deterioration and other environmental problems have become increasingly prominent in rural areas. These environmental concerns have caused irreparable losses to production and human health [1,2]. However, it is extremely difficult to ease the pressure on the rural environment. Rural environmental issues are even more challenging to address than urban ones because the former is confronted with not only the ineffectiveness of public goods, but also the slow development of the rural environmental market with its own specificity. Therefore, given the ineffective rural environmental governance, either government-led or private-sector-initiated, it is vital to promote the proactive participation of rural residents in pro-environmental behavior (PEB). PEB is considered a social cooperation that requires individuals to sacrifice part of their interests for the greater collective good [3]. In this case, a rational self-interested economic person is usually reluctant to spontaneously take this cooperative action, PEB [4]. Nevertheless, researchers have provided a wealth of empirical evidence on the influencing factors of individuals’ PEB. Most of these studied factors are objective ones [5,6,7], including individual objective factors and social objective factors, while subjective factors are scarcely explored. With the rise of behavioral economics, researchers have increasingly recognized that subjective factors, such as emotions, play an essential role in individual’s behavioral decision making [8,9]. Moreover, it has been argued that subjective factors are more likely than objective factors to promote individuals’ pro-social behavior in terms of enhancing social cooperation, and the behavioral change driven by subjective factors is stable in long term [10]. Studies have shown that subjective factors also play an important role in promoting environmental sustainability [11,12]. Therefore, it is necessary to focus on subjective factors when motivating rural residents to develop PEB voluntarily.

Subjective well-being is the comprehensive state of an individual’s emotions and satisfaction based on the individual’s conditions and has a high marginal utility for the individual [13]. With the quiet rise of happiness economics, economists have realized that subjective well-being or happiness is a more justified proxy than income to measure people’s level of well-being [14]. Numerous studies have shown that subjective well-being plays a non-negligible role in promoting pro-social behavior [15,16]. Subjective well-being helps to motivate individuals’ goodwill, enhance altruism and promote individuals to treat others in a more friendly way [17,18,19]. This assertion has been supported by extensive research. For example, individuals with higher subjective well-being show less hostility towards others, higher social adaptation and communication skills, and more cooperative behaviors [20,21]. To sum up, subjective well-being has become a key factor for individuals participating in social cooperation [15,20].

PEB, a type of pro-social behavior, requires individuals to voluntarily participate in social cooperation to benefit the communities or other individuals. Theoretically, subjective well-being should have a positive impact on PEB. However, empirical evidence on the relationship between subjective well-being and PEB is still scarce. Most previous literature on the relationship, based on emotion-inducing experiments, found that positive emotions (subjective well-being) help individuals to participate in PEB [22,23]. However, the existing literature has not explored the influencing mechanism of subjective well-being on PEB, which requires further systematic research [24]. Meanwhile, the existing literature also ignores the impact of subjective well-being on PEB in specific rural contexts. It is worth noting that the rural environment system is distinct from the urban one. Urban environmental protection issues are mainly purely public issues, that is, the beneficiary of environmental protection is the entire urban society. However, rural environmental issues contain certain private interest attributes in addition to the public interest attributes. For example, garbage in the village only affects the villagers’ personal impression or feeling, though the application of chemical fertilizers with a peculiar smell or the discharge of livestock manure affect the air and water quality of the whole village. The environmental protection behavior of rural residents is not as merely altruistic as that of urban residents; instead, it may represent self-interest and reciprocity to a certain degree. Therefore, it is necessary to specifically analyze how subjective well-being affects the PEB of rural residents.

Based on the 2013 Chinese General Social Survey (CGSS2013) data, this study systematically examines the influence of subjective well-being on the PEB of rural residents and explores the mechanism of such influence. Following Stern’s [25] classification of behavior, this study divides PEB into two types, namely, the private-sphere PEB and the public-sphere PEB. The private-sphere PEB refers to environmental protection actions that individuals take to benefit themselves or their families and are closely related to daily life, such as not littering; the public-sphere PEB refers to individuals’ voluntary participation in environmental protection or active participation in addressing environmental problems, such as donating money to environmental protection and participating in collective afforestation. Therefore, concerning the beneficiaries of PEB, the private-sphere one mainly concentrates on individuals’ families or the communities they live in, whereas the public-sphere one benefits broad groups, going beyond oneself, neighbors and friends to cover even strangers. In this regard, despite individuals’ PEB being generally regarded as pro-social altruistic [26,27], it should be understood from two perspectives: the public-sphere one is more altruistic, while the private-sphere one is more reciprocal.

This study found that subjective well-being significantly and positively impacts both the private-sphere PEB with reciprocity and private-sphere PEB with altruistic properties. This result is robust to various internal validity checks. To verify the mechanism underlying the relationship between subjective well-being and PEB, this study examines the moderating effects of reciprocity and altruism on the relationship, using individuals’ social frequency and environmental knowledge to represent reciprocity and altruism, respectively. Our empirical results suggest that subjective well-being influences rural residents’ PEB based on reciprocal and altruistic motivations. In other words, subjective well-being promotes PEB by enhancing social reciprocity between rural residents and others and increasing their fraternity and altruism. Since the effect of subjective well-being on PEB may vary across different groups, this study adopted a microscopic heterogeneity analysis. We found that the positive effect of subjective well-being on the PEB of rural residents is more driven by women than by men. Additionally, environmental knowledge moderates the relationship between rural residents’ subjective well-being and PEB. The higher the level of rural residents’ environmental knowledge, the stronger the effect of subjective well-being on PEB.

This study contributes to the existing literature in several ways. First, it theoretically explains and empirically tests whether PEB is affected by subjective well-being in rural contexts, which has been largely ignored in previous studies. As mentioned above, the PEB of rural residents has unique characteristics. Second, this study contributes to a better understanding of the mechanism underlying the influence of rural residents’ subjective well-being on PEB. Previous research on the relationship primarily treats PEB as a whole without distinguishing between different types. This study emphasizes that the private-sphere PEB and public-sphere PEB have different attributes and empirically examines PEB in the two spheres. The mechanistic analysis further validates that subjective well-being promotes PEB based on reciprocity and altruistic motivation. Finally, few studies have paid attention to the heterogenous influence of subjective well-being on PEB among different groups; this study fills the gap by identifying the heterogeneity caused by gender and environmental knowledge.

The rest of this paper is arranged as follows. Section 2 reviews the literature and proposes the conceptual hypotheses. Section 3 presents the research design, including data description, model assumptions and descriptive statistics. Section 4 demonstrates the empirical research results of the influence of subjective well-being on the PEB of rural residents by conducting benchmark regression, robustness testing, mechanism analysis and heterogeneity analysis. Section 5 concludes the study.

## 2. Literature Review

### 2.1. The Influence of Subjective Well-Being on the PEB of Rural Residents

Individuals’ positive psychological emotions, such as being more caring, more sympathetic and happier, can promote pro-social and pro-environmental behaviors [28]. Subjective well-being is particularly important for individual behavioral decision making [29]. Previous experimental economics studies have confirmed that subjective well-being can inhibit retaliatory behaviors and improve pro-social behaviors [30,31]. Game experiments also have shown that the happier the individuals, the higher their tolerance for others’ selfish behaviors and the greater their interest in cooperation [32,33]. In terms of environmental resources, studies have also confirmed the importance of positive psychological factors, such as subjective well-being in reducing resource consumption and maintaining environmental sustainability [34,35,36]. On this basis, other studies have shown that subjective well-being, longevity and sustainable behavior are positively correlated [37]. In other words, individuals with higher subjective well-being have greater environmental awareness, health awareness and care for future generations, so they are willing to improve their quality of life by engaging more in sustainable behaviors.

In rural areas where the unified environmental management service provided by the government is lacking, whether the local environment is clean primarily affects rural residents themselves. Therefore, it is believed that rural residents’ subjective well-being influences not only their public-sphere PEB with pure altruism, but also the private-sphere PEB with self-interest and reciprocity.

### 2.2. Mechanisms: Reciprocity and Altruism

Subjective well-being can affect the PEB of rural residents in two ways: reciprocity and altruism.

First, an important mechanism by which subjective well-being influences rural residents’ PEB is to shape the reciprocity level of rural residents in the region. The happier the individuals, the more positive emotions they have, and thus the more frequently they participate in social gatherings to enhance their social capital [38,39] that can effectively promote individual participation in collective cooperation, such as PEB [40]. Social capital is particularly important for rural residents. Compared with cities, a rural society is a more traditional “acquaintance society” with strong shared memories and close personal relationships based on kinship and geographic proximity. People establish connections through these personal relationships and gradually form a relationship network. For example, in China’s rural “acquaintance society”, the relationship between people is a network that maintains renqing (personal feelings) and interpersonal communications. In this context, high levels of social capital are conducive to promoting cooperation by enhancing information sharing and reducing cooperation risks to achieve mutual benefits [41]. Therefore, a higher subjective well-being helps rural residents to achieve reciprocity with others and thus promote PEB.

Secondly, subjective well-being, a positive subjective feeling, can also increase rural residents’ fraternity and altruism. Psychological studies have found that happiness is positively related to individuals’ good characteristics, such as benevolence and justice [42]. Compared with sad emotions, positive and optimistic emotions are more conducive for individuals to help others and develop pro-social behaviors [43]. Extending to environmental issues, a more philanthropic and altruistic individual is shown to have a higher level of environmental attention, which is often used as a direct or indirect measure of PEB [44,45]. A person with more subjective well-being may be more willing to sacrifice income to protect the environment because they are more generous [46]. In a nutshell, the more positive the rural residents’ subjective feelings, the higher their benevolence and environmental awareness level and the stronger their willingness to adopt PEB.

### 2.3. Moderators: Gender and Environmental Knowledge

Some factors are expected to moderate the relationship between subjective well-being and PEB.

One of these factors is gender. Studies have shown that women generally show higher levels of attention to and engagement in family-oriented PEB, while men are likely to pay more attention to and participate more frequently in economic behaviors [47,48]. This is related to the traditional theory of gender socialization and the resulting division of gender roles [49,50]. According to this theory, socialization in early childhood predisposes women to the role of “caregiver”. Relative to men, women display a worldview that is more concerned with life, health and relationships and empathy for the feelings and needs of others. In this sense, the behavior of women with a “maternal mindset” is more susceptible to emotional influence. Therefore, compared with men, when women’s subjective well-being is higher, they are more likely to stimulate their fraternity and altruism, and then engage in more PEB.

Second, environmental knowledge may also moderate between subjective well-being and PEB. Increased environmental knowledge can deepen individuals’ understanding of environmental issues and thus promote their environmental concern and responsibility [51,52] and PEB [53]. Moreover, it has been found that environment knowledge can promote the relationship between intrinsic perception, such as the perceived green value, and environmental intentions [54]. Therefore, we believe that the promotion effect of subjective well-being on rural residents is stronger when the individual’s environmental knowledge level is higher.

### 2.4. Theoretical Framework

**Figure 1:**
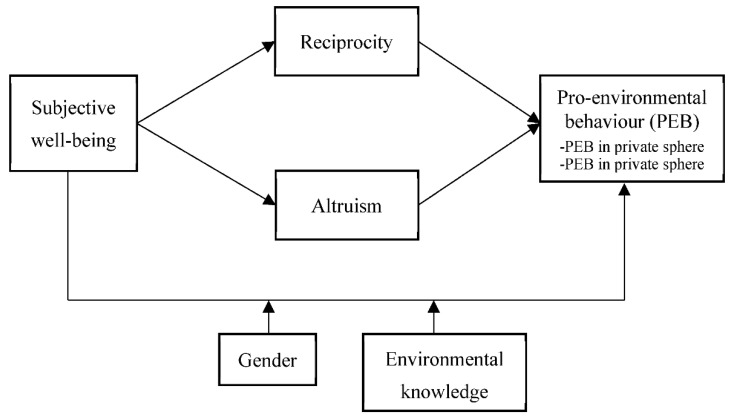
The theoretical framework.

Based on the above analysis, the theoretical framework of this paper was constructed (Figure 1). This framework explains how rural residents’ subjective well-being affects their PEB. Specifically, subjective well-being promotes PEB, both the private-sphere PEB and public-sphere PEB, mainly through reciprocal and altruistic mechanisms. In addition, gender and environmental knowledge moderate the relationship between rural residents’ subjective well-being and PEB. Specifically, the effect of subjective well-being on PEB is mainly driven by women and individuals with higher environmental knowledge.

## 3. Data Sources and Research Methods

### 3.1. Data Sources

This study was based on the 2013 Chinese General Social Survey (CGSS2013) data. CGSS2013 is China’s first nationwide, comprehensive and continuous large-scale social survey data led by Renmin University of China (Beijing, China). It is the most comprehensive and up-to-date data covering both PEBs and subjective attitudes (e.g., individuals’ perception of happiness) in China, which suits well the aim of the present study. The CGSS2013 data adopts a multi-stage random sampling method. The total sample size is 11,438, including 4217 rural samples, distributed across China’s 100 counties (districts) and five major cities (Beijing, Shanghai, Tianjin, Guangzhou and Shenzhen).

### 3.2. Variable Selection

#### 3.2.1. Dependent Variable: PEB

The dependent variable of this study was PEB. The CGSS2013 questionnaire contains ten questions about individuals’ PEB. Following Stern [25], five classified types of environmental behavior were selected as the private-sphere PEB, including “sorting garbage”, “discussing environmental protection with relatives and friends”, “shopping with own bags or baskets”, “reusing plastic packaging” and “paying attention to environmental issues and environmental information”; another five types were classified as the public-sphere PEB, including “participating in environmental protection activities organized by governments”, “participating in environmental protection activities organized by non-governmental environmental protection organizations”, “paying for the maintenance of forests or green spaces”, “donating money to environmental protection” and “participating in complaints and appeals to resolve environmental issues”. Those private-sphere PEBs may benefit rural residents themselves and their neighbors, that is, these behaviors may be motivated by self-interest or reciprocity as long as altruism. However, the benefits of public sector PEBs are much larger and more altruistic driven.

**Figure 2:**
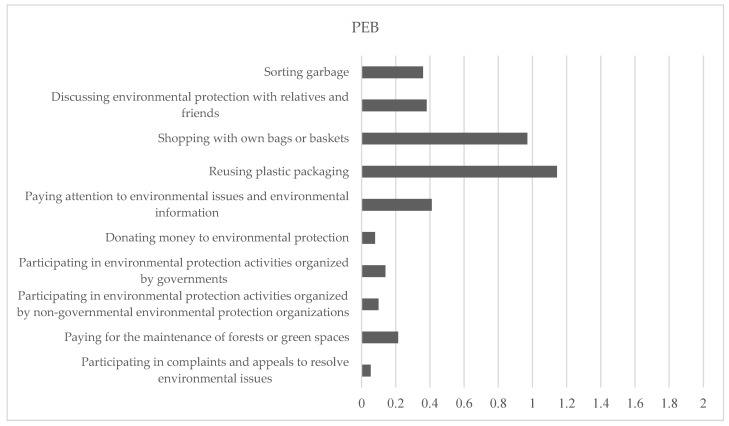
The pro-environmental behavior (PEB) of rural residents.

Figure 2 illustrates the distribution of rural residents’ PEBs by behavior type. The PEBs are ranked in a descending order of participation frequency: reusing plastic packaging, shopping with own bags or baskets, paying attention to environmental issues and environmental information, discussing environmental issues with relatives and friends, sorting garbage, paying for the maintenance of forests or green spaces, participating in environmental protection activities organized by governments, participating in environmental protection activities organized by non-governmental environmental protection organizations, donating money to environmental protection, and participating in complaints and appeals to resolve environmental issues. Overall, rural residents obviously adopted more PEBs in the private sphere (ranking the top five) than in the public sphere (ranking the lowest five).

#### 3.2.2. Key Independent Variable: Subjective Well-Being

**Figure 3:**
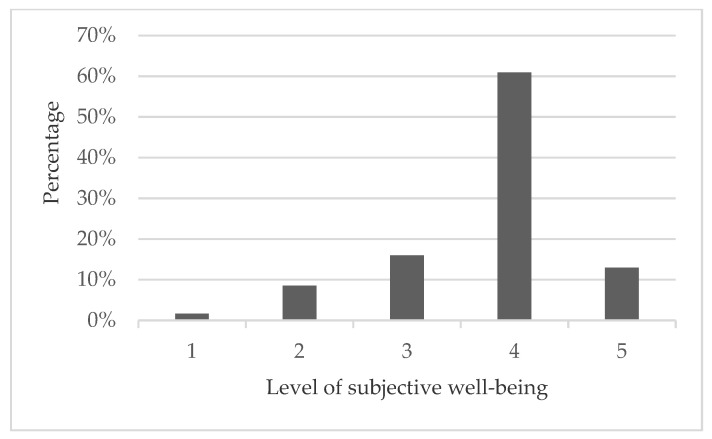
Subjective well-being of rural residents.

According to the research on subjective well-being by Kahneman et al. [55] [43], the most frequently asked questions to measure subjective well-being are: “All things considered, how satisfied are you with your life as a whole?” and “In general, do you think you are happy these days?” This study used the question “In general, do you think your life is happy?” to represent the subjective well-being of rural residents, measured on a 5-point Likert scale ranging from 1 (very unhappy) to 5 (very happy). As shown in Figure 3, 12.96% of rural residents were very happy, 60.89% were happy, 15.99% were neither happy nor unhappy, 8.49% were unhappy, and 1.67% were very unhappy. In general, most rural residents showed a relatively high happiness index.

#### 3.2.3. Mediating Variables: Reciprocity and Altruism

This paper used the closeness of contact between rural residents and their relatives and friends as a proxy variable to measure rural residents’ social interaction and reciprocity with others. The CGSS2013 questionnaire asks the following question to collect information on social interaction: “What is the closeness degree of contact and connection between you and your relatives and friends?” The answers are measured on a 5-point Likert scale.

In the study, the proxy variable for altruism was whether individuals are concerned about air pollution, water pollution, noise pollution, industrial waste pollution, domestic waste pollution, lack of green space, destruction of forest vegetation, degradation of cultivated land quality, shortage of freshwater resources, food pollution, desertification and reduction in wildlife. Individuals who are more concerned about environmental issues tend to have more fraternity and altruism.

#### 3.2.4. Control Variables

According to the existing literature, environmental cognition, personal characteristics and family characteristics impact the PEB of rural residents. This study controlled the following variables: environmental cognitive variable (environmental knowledge), individual demographic characteristics (e.g., age, gender, health status, religious belief, political identity, education level, social network, social status and expectation of social class) and family characteristics variables (e.g., family income, marriage condition, number of children and number of properties). As is shown is Table 1, the above variables are discussed in detail.

### 3.3. Empirical Strategy

To investigate the influence of subjective well-being on the PEB of rural residents, this study employed a model with rural residents’ PEB as the dependent variable, their subjective well-being as the core explanatory variable and their environmental cognitive, individual and family characteristics as the control variables. Since the dependent variable is ordinal, this study adopted an ordered probit model as

(1) $$PEB_{i}=a_{0}+a_{1}Happy_{i}+\sum_{n=1}a_{2}D_{ni}+\varepsilon_{i}.$$

where $PEB_{i}$ is the PEB of the ith rural resident, which is calculated by summing up the frequency of all the ten PEBs. Accordingly, private-sphere PEB and public-sphere PEB are calculated by summing up the frequency of the five private-sphere PEBs and five public-sphere PEBs, respectively. $Happy_{i}$ is the subjective well-being of the ith rural resident. $D_{ni}$ represents control variables, such as the individual, family and regional characteristics, of the ith rural resident and $\varepsilon_{i}$ is an error term.

This identification strategy may suffer from two problems. The first one is an endogeneity problem that may lead to biased or even inconsistent parameter estimation, as reverse causality exists where subjective well-being affects the PEB of rural resident. On the one hand, the happier individuals may be more altruistic and thus participate more in PEB than the less happy ones; on the other hand, PEB may increase subjective well-being, with those who adopt PEB developing a happy mentality of “a little fragrance always clings to the hand that gives the roses”. To solve this endogeneity problem, this study further employed an instrumental variable (IV) analysis based on benchmark regression. The IVs should be related to the core explanatory variable, namely, subjective well-being, but not directly affect the dependent variable, namely, the PEB of rural residents. This study then chose the frequency of individuals’ recreational activities, such as listening to music in their spare time as an IV for subjective well-being. Recreational activities increase rural residents’ happiness, promoting subjective well-being, but they are not related to PEB. In this regard, the frequency of such recreational activities satisfies the instrumental variable selection criteria. The ERM model was selected for the estimation, referring to the practice of Roodman and Botezat [56,57]. The ERM model, which can effectively avoid the endogeneity problem, is suitable for both categorical and continuous endogenous explanatory variables. Specifically, since both the endogenous independent variable and dependent variable (i.e., PEB) are ordinal, this study used the extended oprobit (eoprobit) model for estimation.

The second problem is robustness issues, because the results we obtained from the above estimate cannot be considered reliable and credible unless the conclusion remains unchanged under changing conditions. In this study, several factors may affect the robustness of the empirical results. First, the measurement of the core explanatory variable subjective well-being may cause a robustness problem and thus the robustness analysis was conducted using a different measurement. Second, the robustness problem caused by the sample was tested mainly by using another sample. Finally, the robustness problem caused by the model estimation was tested mainly by adjusting the estimation method.

Next, to examine the mechanism (i.e., reciprocity and altruism) by which subjective well-being affects the PEB of rural residents, the mediating effect model was incorporated into Model (1), constructing the following Models:

(2) $$Mediate_{i}=\beta_{0}+\beta_{1}Happy_{i}+\sum_{n=1}\beta_{2}F_{ni}+\lambda_{i}$$

(3) $$\;\;\;\;\;\;\;\;\;\;\;PEB_{i}=\gamma_{0}+\gamma_{1}Mediate_{i}+\gamma_{2}Happy_{i}+\sum_{n=1}\gamma_{3}G_{ni}+\mu_{i}$$

where $Mediate_{i}$, the mediating variable, represents the frequency of engaging in social interactions and altruism of the ith rural residents. This study examined the mediating effects of social interactions and altruism separately. $PEB_{i}$, the dependent variable, is the PEB of the ith rural residents. $Happy_{i}$, the independent variable, is the subjective well-being of the ith rural resident. $F_{ni}$ and $G_{ni}$ represent control variables, such as the environmental cognitive, individual, family and regional characteristics of the ith rural resident. $\lambda_{i}$ and $\mu_{i}$ are error terms. Specifically, in the case that $a_{1}$ is significant, the mediating effect is established if $\beta_{1}$ and $\gamma_{1}$ are both significant at the same time. With the mediating effect established, significant $\gamma_{2}$ and insignificant $\gamma_{2}$ mean that $Mediate_{i}$ is a partial mediating variable and a complete mediating variable, respectively.

In addition, we conducted a heterogeneity analysis to examine whether the effect of subjective well-being on PEB of rural residents differed across different groups. First, we used grouped regression to examine the impact of subjective well-being on the PEB of rural residents of different genders. Secondly, to examine the impact of subjective well-being on the PEB of rural residents with different levels of environmental knowledge, an interaction term was added into Model (1), constructing the following Model:

(4) $$PEB_{i}=\delta_{0}+\delta_{1}Happy_{i}+\delta_{2}Kownledge_{i}+\delta_{3}Happy_{i}\times Kownledge_{i}+\sum_{n=1}\delta_{4}H_{ni}+\tau_{i}$$

where $Kownledge_{i}$ is the level of understanding about the environment of the ith rural residents. $Happy_{i}\times Kownledge_{i}$ is the interaction term of subjective well-being and the level of environmental knowledge of the ith rural residents. The focus is on $\delta_{3}$, which is significantly positive if environmental knowledge can promote the effect of subjective well-being on the PEB of rural residents.

## 4. Empirical Analysis

### 4.1. The Influence of Subjective Well-Being on the PEB of Rural Residents

#### 4.1.1. Benchmark Regression

Table 2 shows that subjective well-being positively impacted the PEB of rural residents. In other words, the higher the subjective well-being of rural residents, the higher the frequency of their PEB. The PEB of rural residents were further divided into the private-sphere one and the public-sphere one. The oprobit regression results showed that both the private- and public-sphere PEBs of rural residents were significantly positively influenced by their subjective well-being. The private-sphere PEB is often self-interested and reciprocal. The higher the subjective well-being of rural residents, the more likely they adopt PEB in daily life. Moreover, increased subjective well-being helps to strengthen rural residents’ “benevolence” and promote their PEB in the public sphere. Subjective well-being reflects the satisfaction and pleasure of rural residents in their daily life. Rural residents with higher subjective well-being are more satisfied with their living conditions and more willing to improve their life quality, and thus they are more intended to protect the environment to maintain or improve happiness.

Control variables that had significant impacts on rural residents’ PEB include environmental knowledge, social network, gender, education, political identity, health, social class, expectation of social class and the number of real estate properties.

#### 4.1.2. Endogenous Analysis

Given the endogeneity between subjective well-being and the PEB of rural residents, this study used the ERM model, and the results are shown in Table 3. The Durbin–Wu–Hausman statistic was significant, indicating an endogeneity problem. In addition, the F-value of the first-stage joint significance test was greater than 10, indicating that there was no problem of weak instrumental variables. Table 3 shows that after the introduction of IV, subjective well-being still positively influenced the PEB of rural residents in both the private and public spheres. These results are consistent with those of the benchmark regression shown in Table 2, thus indicating their reliability.

#### 4.1.3. Robustness Check

We then turned to the robustness analysis to test the reliability of the effect of subjective well-being on PEB. First, we re-defined subjective well-being. In Table 2, the answer to the question “In general, do you think your life is happy?” was used to describe individuals’ subjective well-being. This paper replaced the independent variable with a substitution one—individuals’ perception of a comfortable life. This substitution variable was derived from the responses to the statement “I live a comfortable life, and there are not many things to worry about” in the CGSS2013 questionnaire. The answer was measured on a 4-point Likert scale, namely, “strongly disagree”, “disagree”, “agree” and “strongly agree”. Individuals’ perception of a comfortable life was then used to measure the individual subjective well-being, and estimate Model 1 again. Panel A in Table 4 shows that subjective well-being still had a significantly positive impact on the PEB of rural residents and the positive effect was identified in both private-sphere PEB and public-sphere PEB. This was generally consistent with the estimation results in Table 2. This means that the research results were still robust when the independent variable was changed.

Secondly, we re-estimated Model 1 by changing the sample. A potential criticism is related to the sample of rural residents, as it includes those with rural *hukou* (registration of residence) and a small number of rural residents with urban *hukou* who may have different behavior patterns. To better understand the impact of subjective well-being on the PEB of genuine rural residents, that is, rural residents with rural *hukou*, this study re-estimated the findings in Table 2 using a sample that excludes those rural residents with urban *hukou*. As is shown in panel B, the results still showed that rural residents with a higher subjective well-being were more inclined to participate in PEB in both the private and public spheres, which was consistent with the results of the benchmark regression. The findings were therefore representative and robust.

Thirdly, the model estimation was tested by adjusting the estimation method. PEB is the self-selection behavior of rural residents, which may cause a self-selection bias-related endogeneity problem to previous models. The propensity score matching (PSM) method can effectively address this problem. Panel C shows the impact of subjective well-being on the PEB of rural residents under three different matching methods. Although a variety of matching methods were used, the influence direction and degree of subjective well-being on the PEB of rural residents were basically the same; the ATT value reflected the fact that rural residents with a higher subjective well-being adopted more PEBs. This was consistent with the estimation results in Table 2, which further confirmed the robustness of the estimation results.

### 4.2. The Mechanism by Which Subjective Well-Being Affects PEB

Theoretically, subjective well-being may affect individuals’ PEB through reciprocity and altruism. This study empirically verified the mechanism between subjective well-being and PEB.

Panel A in Table 5 shows the mediating effect of reciprocity in the relationship between subjective well-being and the PEB of rural residents. As shown in columns (1) and (2), subjective well-being had a significantly positive impact on the PEB of rural residents, under both the oprobit and eoprobit regression models. Additionally, the regression results of oprobit in column (3) and eoprobit in column (4) show that subjective well-being had a significantly positive impact on social interaction. In other words, subjective well-being helped to enhance the level of social interaction and reciprocity between rural residents and others. In addition, the oprobit regression results in column (5) and eoprobit results in column (6) both indicate that, after the introduction of the social interaction variable, subjective well-being still had a significantly positive impact on the PEB of rural residents, and social interaction per se significantly and positively influenced such behavior. According to the test process of the mediating effect constructed in the previous models (Equations (1)–(3)), it is reasonable to conclude that social interaction partially mediated the relationship between subjective well-being and the PEB of rural residents. To test the robustness of the results, this paper used the Sobel test and the generalized structural equation modelling (GSEM) to explore further the mediating effect of social interaction in the influence of subjective well-being on the PEB of rural residents. The Sobel test *p*-value was less than 0.05, and the mediating effect accounted for 24.99% of the total effect, confirming the mediating effect of social interaction. The results of GSEM show that the indirect effect was 0.080, a small coefficient but with a significant *p*-value, verifying the mediating effect; the total effect was 0.314, which was also significant; the mediating effect accounted for 25.48% of the total effect. Therefore, all the results indicate that reciprocity is indeed an essential mechanism by which rural residents’ high subjective well-being transforms into PEB.

Similarly, Panel B shows the mediating effect of altruism in the relationship between subjective well-being and the PEB of rural residents. It can be seen that environmental-related altruism partially mediated the relationship between subjective well-being and the PEB of rural residents. The Sobel test and the generalized structural equation modelling (GSEM) also verified the existence of the partial mediating effect of environmental-related altruism, and calculated that the mediating effect accounted for 25% of the total effect. In a nutshell, these results mean that altruism is also an essential mechanism by which rural residents’ high subjective well-being transforms into PEB.

### 4.3. A Further Analysis

Next, the heterogeneity of the empirical results was explored. The effect of subjective well-being on the PEB of rural residents may differ by gender and environmental knowledge. The discussion and analysis of the heterogeneity can more effectively clarify the mechanism that transforms subjective well-being to PEB, thus facilitating the formulation of well-targeted environmental policies.

#### 4.3.1. Heterogeneity Analysis by Gender

This study examined whether the influence of subjective well-being on the PEB of rural residents differs between genders. As shown in Table 6, the positive effect of subjective well-being on the PEB of rural residents was mainly driven by women. Specifically, such influence was much stronger in the female sample than in the male sample. This is because, as noted above, women are more compassionate and altruistic than men, and thus they are more willing to participate in PEB, which is altruistic and requires sacrificing individual interests for the collective good.

#### 4.3.2. Moderating Role of Environmental Knowledge

This study further explored whether environmental knowledge moderates the effect of subjective well-being on the PEB of rural residents by integrating an interaction variable “environmental knowledge *×* subjective well-being”. Environmental knowledge measures the individuals’ mastery of environmental knowledge.

Table 7 shows that the moderating effect of the interaction between subjective well-being and environmental knowledge was positive, indicating that, when rural residents’ environmental knowledge increased, the positive effect of subjective well-being on PEB significantly increased. Specifically, the positive moderating effect of environmental knowledge was identified in the private sphere.

## 5. Discussion

This paper focused on the impact of subjective well-being on the PEB of rural residents. The empirical results show that subjective well-being promoted PEB in both the public and private spheres. This means that subjective well-being reflects not only rural residents’ purely altruistic motives to participate in collective cooperation, but also their reciprocal motives. On this basis, this study further explored the mechanism by which subjective well-being influences the PEB of rural residents and found that subjective well-being influences their PEB by reciprocity and altruism. Individuals with a higher subjective well-being tend to have more reciprocity with others and more altruism and thus promoted their PEB. The heterogeneity analysis yielded two important results. First, the influence of subjective well-being on the PEB of rural residents was significantly positive in the female group, but it had no significant effect in the male group. This finding is consistent with the theory of gender socialization and the resulting division of gender roles [34,35]. Secondly, subjective well-being was more likely to promote PEB for individuals with a high environmental knowledge.

### 5.1. Theoretical Contributions

This study has made the following contributions. First, it complements the research on the influencing factors of individual PEB. Previous research has provided rich empirical evidence on the objective influencing factors of individual PEB, including socio-demographic characteristics (e.g., gender, age, location, education level and income) and social objective factors, such as social networks [5], government regulation [6] and social supervision [7]. However, few studies have focused on the subjective factors affecting PEB, the key factor motivating individuals’ spontaneous turn to PEB in the long run. Second, this study contributes to the literature on the effects of subjective well-being. The existing literature mainly focuses on the influencing factors of subjective well-being [58,59,60], but the literature on the effects of well-being has only slowly emerged in recent years [55], such as the influence of subjective well-being on economic growth [61], consumption and saving [62], investment and risk identification [63], immigration intentions [64], employment [65], democratic culture [66], personal income and productivity [67] and the reduction in excessive personal risk-taking [68]. This study adds knowledge to the literature by exploring the effects of subjective well-being on PEB.

### 5.2. Practical Implications

The results of this study have important policy implications for rural environmental governance. Improving well-being is critical for the PEB of rural residents. Increasing rural residents’ subjective well-being is not only an important development goal, but also the starting point and foothold of solving the contradiction between economic development and environmental protection in rural areas. Governments should strive to create a better economic and social environment, bestowing rural residents the expectation of a happy life and thus promoting their adoption of PEB. While cultivating rural residents’ subjective well-being to promote PEB is a long-term task, the short-term instrumental coping strategy in the face of severe rural environmental problems is environmental protection propaganda. This study found that reciprocity and altruism are the mediating factors between subjective well-being and the PEB of rural residents. Additionally, subjective well-being plays a greater role in promoting the PEB of rural residents with a higher environmental knowledge. Therefore, extensive environmental protection publicity and environmental education will effectively promote the PEB of rural residents. In addition, the frequent interactions among rural residents are conducive to the spread and diffusion of information, knowledge and technology, and thus increase the likelihood of transforming subjective well-being into PEB. Therefore, environmental organizations can partially address rural environmental governance issues by enhancing the rural residents’ social network (e.g., discussing with friends, neighbors and colleagues about what to do for the environment, how and why). Finally, given that the influence of subjective well-being on the PEB of rural residents is mainly driven by female groups, environmental protection organizations can give priority to those population groups when implementing environmental protection activities and policies, thus encouraging them to lead others to adopt PEB.

### 5.3. Limitations and Future Research

Based on the CGSS2013 data, this study found that improving the subjective well-being of rural residents can help to promote their PEB. This result reveals that subjective factors, such as subjective well-being, play an unignorable role in promoting individual PEB in the face of environmental governance dilemmas. We call for more future research on the influence of subjective factors, such as the perception of fairness, trust and pursuit of reputation, on PEB and broader pro-social behavior. It seems to be an exciting and effective way to explore the issue of environmental governance from the perspective of subjective factors. Inevitably, this study suffers from some limitations. First, this study is embedded in the rural context, and thus the results may differ from those retrieved from the urban context. Future research can further examine the effect of urban residents’ subjective well-being on their PEB and make urban-rural comparisons to deepen the understanding of this issue. Second, this study is based on the CGSS data in 2013. Although the CGSS data has been updated to 2017, it is a pity that the questionnaires after 2013 did not simultaneously collect information on PEB and subjective well-being. Therefore, while it is reasonable for this study to use CGSS2013 data, we call for future research to use updated data to validate the model results of this study. Third, this study explored the causal effect of subjective well-being on PEB, which inevitably suffers from an endogeneity problem. The effects of the endogeneity problem may not be completely excluded, though the instrumental variable method and a series of robustness tests were used to address endogeneity and verify the reliability of the results. Future studies can use longitudinal panel data to verify the results further.

## 6. Conclusions

Given the importance of rural residents’ spontaneous PEB and the positive role of subjective well-being in influencing individual behaviors, this study empirically examined the influence of rural residents’ subjective well-being on their PEB and the mechanism underlying the influence based on CGSS data, which has been largely ignored by previous research. Borrowing the classification of Stern [13], this study divided PEB into two categories, namely, the private-sphere PEB with reciprocal attributes and the public-sphere PEB with altruistic properties. Then, it revealed the different effects of subjective well-being on the two categories and their mechanisms. This study expanded existing research by examining the possible heterogeneous effects of subjective well-being on PEB among different groups.

This study found that subjective well-being promotes rural residents’ participation in social cooperative PEB in both the private and public spheres. This result passed a series of robustness tests, indicating that the estimation results are reliable. The results also suggest that subjective well-being influences rural residents through not only reciprocal motives but also altruistic motives. Further mechanistic tests proved that subjective well-being promotes PEB by enhancing rural residents’ interaction and reciprocity with others and altruism. In this sense, rural residents with higher levels of subjective well-being tend to have more connections with others for mutual benefit and have more good qualities, such as fraternity and altruism. Moreover, this study found that the effect of rural residents’ subjective well-being on their PEB is more driven by women than by men. This conclusion is consistent with the theory of gender socialization and the resulting division of gender roles [49]. We also noticed that environmental knowledge could moderate the relationship between subjective well-being and PEB. The higher the level of the rural residents’ environmental knowledge, the stronger the effect of subjective well-being on PEB, especially the private-sphere PEB.

## Figures and Tables

**Table 1:** Descriptive statistics.

Variable	Definition	Mean	SD	Min	p50	Max
**Dependent variable**						
Private-sphere PEB	Sorting garbage (1–3)	1.360	0.604	1.000	1.000	3.000
	Discuss environmental issues with relatives and friends (1–3)	1.381	0.551	1.000	1.000	3.000
	Shopping with own bags or baskets (1–3)	1.970	0.790	1.000	2.000	3.000
	Reusing plastic packaging (1–3)	2.144	0.799	1.000	2.000	3.000
	Paying attention to environmental issues and environmental information (1–3)	1.411	0.607	1.000	1.000	3.000
Public-sphere PEB	Donating money to environmental protection (1–3)	1.079	0.286	1.000	1.000	2.000
	Participating in environmental protection activities organized by governments (1–3)	1.140	0.394	1.000	1.000	3.000
	Participating in environmental protection activities organized by non-governmental environmental protection organizations	1.099	0.338	1.000	1.000	3.000
	Paying for the maintenance of forests or green spaces (1–3)	1.214	0.516	1.000	1.000	3.000
	Participating in complaints and appeals that resolve environmental issues (1–3)	1.053	0.249	1.000	1.000	2.000
**Main independent variable**						
Subject well-being	Level of subjective well-being (1–5)	3.750	0.847	1.000	4.000	5.000
**Control variable**						
Environmental knowledge ^1^	The level of understanding about environmental knowledge (0–1)	0.721	0.449	0.000	1.000	1.000
Age	Actual value (Year)	50.816	15.410	19.000	50.000	82.000
Gender	Male = 1, female = 0	0.497	0.500	0.000	0.000	1.000
Health	Self-perception of health status (1–5)	3.532	1.167	1.000	4.000	5.000
Political identity	CPC (Communist Party of China) member =1, non-CPC member =0	0.051	0.220	0.000	0.000	1.000
Education level	Never went to school = 1, primary school = 2, junior high school = 3, high school = 4, university and above = 5	2.347	1.004	1.000	2.000	5.000
Religion ^2^	Have a religious belief = 1, have no religious belief = 0	0.116	0.320	0.000	0.000	1.000
Social network	Frequency of contact with relatives and friends (1–5)	3.422	0.865	1.000	4.000	5.000
Social class	Self-perception of social class (1–10, top = 10, bottom = 1)	4.080	1.686	1.000	4.000	9.000
Expectation of social class	Self-perception of the social class in 10 years (1–10, top = 10, bottom = 1)	5.099	2.026	1.000	5.000	10.000
Family income	Natural logarithm of family income for one year	8.789	3.365	0.000	9.904	11.918
Marriage	Married = 1, others = 0	0.940	0.237	0.000	1.000	1.000
Number of children	The number of children in the family	2.142	1.327	0.000	2.000	6.000
Number of properties	The number of real estate properties owned by the family (0–10)	1.111	0.441	0.000	1.000	3.000

^1^ Answer an environmental knowledge question, correct = 1, wrong or do not know = 0. ^2^ Religious beliefs include Buddhism, Catholicism, Christianity, Islam and Judaism and so on: have any religious belief = 1, have no religious belief = 0.

**Table 2:** The influence of subject well-being on the PEB of rural residents.

	(1)	(2)	(3)
	Overall PEB	Private-Sphere PEB	Public-Sphere PEB
Subjective well-being	0.072 ***	0.071 ***	0.058 **
	(0.021)	(0.021)	(0.028)
Environmental awareness			
Environmental knowledge	0.360 ***	0.352 ***	0.249 ***
	(0.038)	(0.038)	(0.052)
Demographics			
Age	−0.003 *	−0.003 *	−0.000
	(0.002)	(0.002)	(0.002)
Male	−0.033	−0.077 **	0.106 **
	(0.036)	(0.036)	(0.046)
Health	0.014	0.004	0.036 *
	(0.016)	(0.017)	(0.021)
CPC member	0.350 ***	0.222 ***	0.432 ***
	(0.083)	(0.078)	(0.089)
Education level (never went to school = control)			
Primary school	0.035	0.050	0.041
	(0.045)	(0.045)	(0.066)
Junior high school	0.205 ***	0.210 ***	0.186 **
	(0.053)	(0.053)	(0.073)
High school	0.381 ***	0.341 ***	0.462 ***
	(0.077)	(0.077)	(0.090)
University and above	0.786 ***	0.705 ***	0.767 ***
	(0.117)	(0.115)	(0.134)
Religion	0.203 ***	0.174 ***	0.165 **
	(0.056)	(0.055)	(0.075)
Social network	0.068 ***	0.055 ***	0.096 ***
	(0.020)	(0.020)	(0.026)
Social class	−0.054 ***	−0.050 ***	−0.034 *
	(0.015)	(0.016)	(0.019)
Expectation of social class	0.063 ***	0.062 ***	0.033 **
	(0.013)	(0.013)	(0.016)
Family characteristics			
Family income	0.011 **	0.014 ***	0.002
	(0.006)	(0.005)	(0.007)
Marriage	0.005	−0.021	0.069
	(0.082)	(0.081)	(0.097)
Number of children	−0.020	−0.011	−0.051 **
	(0.016)	(0.016)	(0.022)
Number of properties	0.051	0.039	0.062
	(0.037)	(0.038)	(0.046)
Region dummy variable	yes	yes	yes
Obs.	4055	4066	4068
Pseudo R^2^	0.034	0.032	0.066

Note: *** *p <* 0.01, ** *p <* 0.05, * *p <* 0.10. Standard errors are in parenthesis.

**Table 3:** The influence of subject well-being on PEB: endogeneity analysis.

	(1)	(2)	(3)
	Overall PEB	Private-Sphere PEB	Public-Sphere PEB
Subjective well-being	1.046 ***	0.969 ***	1.072 ***
	(0.042)	(0.059)	(0.037)
Control variables	yes	yes	yes
Region dummy variable	yes	yes	yes
DWH test	84.969 ***	66.952 ***	49.302 ***
F-stat.	13.583 ***	13.710 ***	14.010 ***
Under-identification test	13.882 ***	14.020 ***	14.290 ***
Obs.	3998	4009	4009

Note: *** *p <* 0.01. Standard errors are in parenthesis. “Yes” is added to indicate a series of control variables being controlled, such as environmental knowledge, age, gender, health status, political identity, education level, religion, social network, social class, expectation of social class, family income, marriage, number of children, number of properties and region dummy variable.

**Table 4:** Robustness check.

	(1)	(2)	(3)
	Overall PEB	Private-Sphere PEB	Public-Sphere PEB
*Panel A: Substitution of the independent variable*			
Subjective well-being	0.105 ***	0.101 ***	0.081 ***
	(0.020)	(0.020)	(0.024)
Control variables	yes	yes	yes
Region dummy variable	yes	yes	yes
Obs.	4023	4034	4034
*Panel B: Change of sample*			
Subjective well-being	0.073 ***	0.073 ***	0.059 **
	(0.022)	(0.022)	(0.029)
Control variables	yes	yes	yes
Region dummy variable	yes	yes	yes
Obs.	3807	3818	3819
*Panel C: Substitution of the estimation method*			
Nearest neighbour matching	0.585 ***	0.421 ***	0.136 **
Radius matching	0.501 ***	0.391 ***	0.107 **
Kernel Matching	0.494 ***	0.383 ***	0.105 **

Note: *** *p <* 0.01, ** *p <* 0.05. Standard errors are in parenthesis. “Yes” is added to indicate a series of control variables being controlled, such as environmental knowledge, age, gender, health status, political identity, education level, religion, social network, social class, expectation of social class, family income, marriage, number of children, number of properties and region dummy variable.

**Table 5:** The test results of the mechanism (reciprocity) by which subjective well-being influences the PEB of rural residents.

	(1)	(2)	(3)	(4)	(5)	(6)
	PEB		Social Network		PEB	
	Oprobit	Eoprobit	Oprobit	Eoprobit	Oprobit	Eoprobit
*Panel A: reciprocity*						
Subjective well-being	0.081 ***	1.057 ***	0.163 ***	0.573 ***	0.073 ***	1.051 ***
	(0.021)	(0.044)	(0.023)	(0.157)	(0.021)	(0.045)
Social interaction					0.067 ***	0.032 ***
					(0.020)	(0.011)
Control variables	yes	yes	yes	yes	yes	yes
Region dummy variable	yes	yes	yes	yes	yes	yes
Obs.	4055	4055	4093	4093	4055	4055
*Panel B: altruism*						
Subjective well-being	0.081 ***	1.057 ***	0.077 ***	0.871 ***	0.069 ***	1.034 ***
	(0.021)	(0.044)	(0.022)	(0.091)	(0.021)	(0.050)
Environmental awareness					0.059 ***	0.031 ***
					(0.005)	(0.005)
Control variables	yes	yes	yes	yes	yes	yes
Region dummy variable	yes	yes	yes	yes	yes	yes
Obs.	4055	4055	4085	4085	4049	4049

Note: *** *p <* 0.01. Standard errors are in parenthesis. “Yes” is added to indicate a series of control variables being controlled, such as environmental knowledge, age, gender, health status, political identity, education level, religion, social network, social class, expectation of social class, family income, marriage, number of children, number of properties and region dummy variable.

**Table 6:** The influence of subjective well-being on the PEB of rural residents by gender.

	(1)	(2)	(3)	(4)	(5)	(6)
	Male			Female		
	Overall PEB	Private-Sphere PEB	Public-Sphere PEB	Overall PEB	Private-Sphere PEB	Public-Sphere PEB
Subjective well-being	0.040	0.049	0.015	0.114 ***	0.101 ***	0.115 ***
	(0.031)	(0.031)	(0.038)	(0.029)	(0.029)	(0.041)
Control variables	yes	yes	yes	yes	yes	yes
Region dummy variable	yes	yes	yes	yes	yes	yes
Obs.	2021	2028	2029	2034	2038	2039
Pseudo R^2^	0.029	0.027	0.068	0.044	0.044	0.065

Note: *** *p <* 0.01. Standard errors are in parenthesis. “Yes” is added to indicate a series of control variables being controlled, such as environmental knowledge, age, gender, health status, political identity, education level, religion, social network, social class, expectation of social class, family income, marriage, number of children, number of properties and region dummy variable.

**Table 7:** The influence of subjective well-being on the PEB of rural residents: the moderating role of environmental knowledge.

	(1)	(2)	(3)
	Overall PEB	Private-Sphere PEB	Public-Sphere PEB
Subjective well-being	0.008	−0.024	0.150
	(0.122)	(0.125)	(0.184)
Subjective well-being × environmental knowledge	0.302 **	0.347 **	0.026
	(0.141)	(0.143)	(0.207)
Environmental knowledge	0.399 ***	0.348 ***	0.448 **
	(0.121)	(0.124)	(0.179)
Control variables	yes	yes	yes
Region dummy variable	yes	yes	yes
Obs.	4081	4092	4094
Pseudo R^2^	0.035	0.032	0.068

Note: *** *p <* 0.01, ** *p <* 0.05. Standard errors are in parenthesis. “Yes” is added to indicate a series of control variables being controlled, such as environmental knowledge, age, gender, health status, political identity, education level, religion, social network, social class, expectation of social class, family income, marriage, number of children, number of properties and region dummy variable.

## Data Availability

The data is available in a publicly accessible repository: http://www.cnsda.org/index.php?r=projects/view&id=93281139 (accessed on 25 January 2020).
